# Supplementary material for: Hospital Variation in Feeding Jejunostomy Policy for Minimally Invasive Esophagectomy: A Nationwide Cohort Study
Source: Nutrients. 2022 Dec 29;15(1):154. doi: 10.3390/nu15010154 (PMC9823823; doi:10.3390/nu15010154)
Supplement: Supplementary file 1 [file nutrients-15-00154-s001.zip › nutrients-2106421-supplementary.pdf]

Hospital	Routine placement	Entrance to abdomen	Fixation to abdominal wall	Type of suture	Anti-rotation suture	Side of anti-rotation suture	Technique of anti-rotation suture	Fixation to outer skin	Starting time of feeding	Feeding protocol	Days post-surgery to full feeding
A	Yes	Laparoscopy	Interrupted suture	Vicryl (braided, dissolvable)	Yes	Distal	Interrupted suture bowel - abdominal wall	Triangle fixation	Morning after surgery	Per protocol by dietician	2
B	Yes	Laparoscopy	Interrupted suture	Vicryl (braided, dissolvable)	Yes	Distal	Interrupted suture bowel - abdominal wall	Direct fixation to skin	No tube feeding unless direct oral feeding is not possible / sufficient	Per protocol by dietician	3
C	Yes	Robot-assisted	Purse string suture	V-loc (or identical)	Yes	Proximal and distal	Interrupted suture caudal; cranial V-Loc	Direct fixation to skin	Morning after surgery	On patient condition	Dependent on defecation
D	Yes	Minilaparotomy (<5 cm)	Purse string suture	PDS (monofilament, dissolvable)	Yes	Proximal and distal	Interrupted suture bowel - abdominal wall	First triangle fixation, afterwards statlock butterfly plaster	Morning after surgery	Day 1 at 500ml/24u	After good defecation, usually day 4
E	Yes	Minilaparotomy (<5 cm)	Purse string suture	Vicryl (braided, dissolvable)	Yes	Proximal and distal	Interrupted suture bowel - abdominal wall	Triangle fixation	Straight after surgery	Per protocol by dietician	3
F	Yes	Laparoscopy	Purse string suture	V-loc (or identical)	No			Triangle fixation	Straight after surgery	On patient condition	5
G	Yes	Through one of the trocart openings a vue	Purse string suture	PDS (monofilament, dissolvable)	No			Triangle fixation	Morning after surgery	On patient condition	2 days, formerly 1 day
H	No	Laparoscopy	Purse string suture	V-loc (or identical)	No			Triangle fixation	Straight after surgery	Per protocol by dietician	No tube feeding
I	Yes	Minilaparotomy (<5 cm)	Purse string suture	Vicryl (braided, dissolvable)	Yes	Distal	Interrupted suture bowel - abdominal wall	Triangle fixation	Morning after surgery	Per protocol by dietician	3
J	Yes	Minilaparotomy (<5 cm)	U-suture on the jejunum, with FJ running through it, attached on four sides of the FJ to the abdominal wall	Vicryl (braided, dissolvable)	No			Triangle fixation	After surgery 10cc NaCl/hour; Morning after surgery start 30cc tube feeding per hour	Day 1 30cc/h. When defecating raise with 15cc/hour per day (maximum 84cc/hour). After good barium swallow X-ray start 1 liter during the night.	5
K	Yes	Laparoscopy	Both purse string and continuous suture	V-loc (or identical)	No			Triangle fixation	Morning after surgery	Per protocol by dietician	2
L	Yes	Minilaparotomy (<5 cm)	Interrupted suture	Vicryl (braided, dissolvable)	Yes	Proximal	Interrupted suture bowel - abdominal wall	Butterfly plaster	Straight after surgery	Per protocol by dietician	2-3 days
M	Yes	Minilaparotomy (<5 cm)	Two purse string sutures to fixate FJ in jejunum and interrupted sutures for fixation to the abdominal wall	Vicryl (braided, dissolvable)	Yes	Proximal and distal	Interrupted suture bowel - abdominal wall	Direct fixation to skin	Straight after surgery	Per protocol by dietician	3

Table S1. Results of survey questionnaire regarding routine placement of feeding jejunostomy, placement techniques and feeding protocol.

Outcome measures	Definition
Overall intra-operative complications	Any Clavien-Dindo grade
Overall postoperative complications	Any Clavien-Dindo grade
Severe postoperative complications	Defined as Clavien-Dindo grade IIIa or higher
30-day or in-hospital mortality	Mortality during primary hospital admission, or in case of discharge within 30-days postoperatively
Chyle leakage	Definition by the ECCG[1]
Anastomotic leakage (radiologically and endoscopically confirmed)	Definition by the ECCG[1]
Pulmonary complications	Definition by the ECCG[1]
Pneumonia	Definition by the ECCG[1]
Wound infections	Definition by the ECCG[1]
Length of hospital stay	Dichotomized around the national median length of hospital stay into 'prolonged admission' and 'short admission', with the exact median added to the 'short admission' group
Prolonged hospital	Hospital stay >30 days
30-day readmissions	Readmission to the hospital within 30 days after surgery
Reinterventions	Either surgical, radiological or endoscopic

1. Esophagectomy Complications Consensus Group. ECCG complication definitions. https://esodata.net/web/complication-definitions

Table S2. Postoperative outcome measures included for analysis .

Variable	Categories
Sex	Male, Female
Age	< 65, 65 – 75, > 75
Preoperative weight loss (kg)	No weight loss, 1 – 5, 6 – 10, > 10, Missing
BMI	< 20, 20 – 25, 26 – 30, > 30, Missing
CCI	0, 1, 2 +, Missing
ASA	1 – 2, 3 +, Missing
Diabetes	Yes, No, Missing
Previous esophageal or gastric surgery	Yes, No, Unknown / Missing
Tumor location	Intrathoracic esophagus, Gastro-esophageal junction, Unknown / Missing
Histology	Adenocarcinoma, Squamous cell carcinoma, Other / Unknown, Missing
Clinical tumor stage	T0 – 2, T3 – 4, Tx, Missing
Clinical node stage	N0, N+, Nx, Missing
Neoadjuvant therapy	None, Chemoradiotherapy, Chemotherapy, Other / Missing
Surgical procedure	Transhiatal, Transthoracic
Anastomotic location	Intrathoracic, Cervical, None / Other / Missing
Hospital volume (the annual total esophagectomy hospital volume was assigned to each patient and thereafter dichotomized into ≤40 or >40)	≤ 40, > 40

Table S3. Variables included in multilevel multivariable regression analyses.

	Patients without FJ N (%)	Patient with FJ N (%)	Total N (%)	P-value (χ^2/Fisher)
Total	305 (100%)	249 (100%)	554 (100%)	
Sex				0.321
Male	224 (73.4%)	192 (77.1%)	416 (75.1%)	
Female	81 (26.6%)	57 (22.9%)	138 (24.9%)	
Age in years				0.227
< 65	125 (41.0%)	88 (35.3%)	213 (38.4%)	
65-75	148 (48.5%)	125 (50.2%)	273 (49.3%)	
> 75	32 (10.5%)	36 (14.5%)	68 (12.3%)	
Preoperative weight loss (kg)				0.232
No weight loss	96 (31.5%)	64 (25.7%)	160 (28.9%)	
1-5	79 (25.9%)	78 (31.3%)	157 (28.3%)	
6-10	74 (24.3%)	54 (21.7%)	128 (23.1%)	
> 10	44 (14.4%)	36 (14.5%)	80 (14.4%)	
Missing	12 (3.9%)	17 (6.8%)	29 (5.2%)	
Body Mass Index (BMI) (kg/m^2)				**0.014**
< 20	13 (4.3%)	18 (7.2%)	31 (5.6%)	
20-25	145 (47.5%)	140 (56.2%)	285 (51.4%)	
26-20	101 (33.1%)	71 (28.5%)	172 (31.0%)	
> 30	44 (14.4%)	19 (7.6%)	63 (11.4%)	
Missing	2 (0.7%)	1 (0.4%)	3 (0.5%)	
Charlson Comorbidity Index				0.521
0	116 (38.0%)	105 (42.2%)	221 (39.9%)	
1	66 (21.6%)	46 (18.5%)	112 (20.2%)	
2 +	113 (37.0%)	98 (39.4%)	211 (38.1%)	
Missing	10 (3.3%)	0 (0%)	10 (1.8%)	
ASA				**0.003**
1-2	208 (68.2%)	135 (54.2%)	343 (61.9%)	
3 +	97 (31.8%)	108 (43.4%)	205 (37.0%)	
Missing	0 (0%)	6 (2.4%)	6 (1.1%)	
Previous esophageal or gastric surgery				0.480
Yes	299 (98.0%)	246 (98.8%)	545 (98.4%)	
No	6 (2.0%)	3 (1.2%)	9 (1.6%)	
Unknown/missing	0 (0%)	0 (0%)	0 (0%)	
Tumor location				0.705
Intrathoracic esophagus	240 (78.7%)	190 (76.3%)	430 (77.6%)	
Gastro-esophageal junction	63 (20.7%)	58 (23.3%)	121 (21.8%)	
Unknown/missing	2 (0.7%)	1 (0.4%)	3 (0.5%)	
Histology				**0.020**
Adenocarcinoma	256 (83.9%)	193 (77.5%)	449 (81.0%)	
Squamous cell carcinoma	37 (12.1%)	52 (20.9%)	89 (16.1%)	
Other/unknown	8 (2.6%)	4 (1.6%)	12 (2.2%)	
Missing	4 (1.3%)	0 (0%)	4 (0.7%)	
Clinical tumor stage				0.642
T0-2	52 (17.0%)	44 (17.7%)	96 (17.3%)	
T3-4	234 (76.7%)	194 (77.9%)	428 (77.3%)	
Tx	19 (6.2%)	11 (4.4%)	30 (5.4%)	
Missing	0 (0%)	0 (0%)	0 (0%)	
Clinical node stage				0.729
N0	116 (38.0%)	101 (40.6%)	217 (39.2%)	
N+	172 (56.4%)	137 (55.0%)	309 (55.8%)	
Nx	17 (5.6%)	11 (4.4%)	28 (5.1%)	
Missing	0 (0%)	0 (0%)	0 (0%)	
Neoadjuvant therapy				**0.035**
None	16 (5.2%)	27 (10.8%)	43 (7.8%)	
Chemoradiotherapy	263 (86.2%)	197 (79.1%)	460 (83.0%)	
Chemotherapy	26 (8.5%)	25 (10.0%)	51 (9.2%)	
Other/missing	0 (0%)	0 (0%)	0 (0%)	
Surgical procedure				**< 0.001**
Transhiatal	14 (4.6%)	53 (21.3%)	67 (12.1%)	

Transthoracic	291 (95.4%)	196 (78.7%)	487 (87.9%)	
Anastomotic location				**< 0.001**
Intrathoracic	234 (76.7%)	137 (55.0%)	371 (67.0%)	
Cervical	71 (23.3%)	112 (45.0%)	183 (33.0%)	
None/other/missing	0 (0%)	0 (0%)	0 (0%)	
Hospital volume (esophageal resections per year)				**0.038**
≤ 40	43 (14.1%)	21 (8.4%)	64 (11.6%)	
> 40	262 (85.9%)	228 (91.6%)	490 (88.4%)	
Missing	43 (14.1%)	21 (8.4%)	64 (11.6%)	

Table S4. Patient, tumor and treatment characteristics of patients without and with feeding jejunostomy (FJ) in hospitals placing feeding jejunostomies in <90% of patients.
